# Supplementary material for: Suppression of Hypoxia-Inducible Factor 1α by Low-Molecular-Weight Heparin Mitigates Ventilation-Induced Diaphragm Dysfunction in a Murine Endotoxemia Model
Source: Int J Mol Sci. 2021 Feb 8;22(4):1702. doi: 10.3390/ijms22041702 (PMC7914863; doi:10.3390/ijms22041702)
Supplement: Supplementary file 1 [file ijms-22-01702-s001.pdf]

Table S1. Physiologic conditions at the beginning and end of ventilation.

	Nonventilated	Nonventilated LPS	V_T 6 ml/kg LPS	V_T 30 ml/kg LPS	V_T 30 ml/kg LPS+HIF-1α	V_T 30 ml/kg LPS+LMWH
PH	7.41±0.07	7.38±0.03	7.36±0.08	7.39±0.08	7.37±0.06	7.39±0.08
PaO2 (mmHg)	98.4±0.3	92.3±0.4	89.7±0.4*	73.4±2.6*	86.2±2.3*	85.6±1.9*
PaCO2 (mmHg)	39.1±0.2	39.8±0.3	38.9±1.3	38.5±1.4	37.6±1.4	37.9±1.5
MAP (mmHg)						
Start	85.6±1.2	83.9±0.5	84.8±1.4	82.5±2.4	84.7±2.2	84.8±2.3
End	85.1±0.4	81.3±0.3	79.2±2.1*	75.4±2.1*	78.3±2.4*	78.4±2.6*
PIP (mmHg)						
Start			15.8±1.3	16.3±1.2	15.7±1.1	15.8±1.6
End			16.9±1.6	17.7±1.8	17.1±1.4	17.3±1.4

At the end of the study period, we obtained data of mean arterial pressure and arterial blood gases from the nonventilated control mice and mice ventilated at a tidal volume of 6 mL/kg or 10 mL/kg for 8 h (n = 10 per group). The normovolemic statuses of mice were maintained by monitoring mean artery pressure. Data are presented as means ± SDs. * indicates that $P < 0.05$ when compared to the nonventilated control mice. HIF = hypoxia-inducible factor; LMWH = low-molecular-weight heparin; LPS = lipopolysaccharide; MAP = mean arterial pressure; PIP = peak inspiratory pressure; V_T = tidal volume.
